# ADVCSO: Adaptive Dynamically Enhanced Variant of Chicken Swarm Optimization for Combinatorial Optimization Problems

**DOI:** 10.3390/biomimetics10050303

**Published:** 2025-05-09

**Authors:** Kunwei Wu, Liangshun Wang, Mingming Liu

**Affiliations:** 1 Mechanical and Electrical Engineering College, Hainan University, Haikou 570228, China; 20223001000@hainanu.edu.cn; 2 Research Institute of Information Technology, Tsinghua University, Beijing 100101, China; 3 School of Mechanical Engineering, Beijing Institute of Technology, Beijing 100081, China; 3120220325@bit.edu.cn

**Keywords:** chicken swarm optimization, good point set elite perturbation initialization, dynamic role allocation mechanism, cosine annealing strategy, hybrid mutation strategy

## Abstract

High-dimensional complex optimization problems are pervasive in engineering and scientific computing, yet conventional algorithms struggle to meet collaborative optimization requirements due to computational complexity. While Chicken Swarm Optimization (CSO) demonstrates an intuitive understanding and straightforward implementation for low-dimensional problems, it suffers from limitations including a low convergence precision, uneven initial solution distribution, and premature convergence. This study proposes an Adaptive Dynamically Enhanced Variant of Chicken Swarm Optimization (ADVCSO) algorithm. First, to address the uneven initial solution distribution in the original algorithm, we design an elite perturbation initialization strategy based on good point sets, combining low-discrepancy sequences with Gaussian perturbations to significantly improve the search space coverage. Second, targeting the exploration–exploitation imbalance caused by fixed role proportions, a dynamic role allocation mechanism is developed, integrating cosine annealing strategies to adaptively regulate flock proportions and update cycles, thereby enhancing exploration efficiency. Finally, to mitigate the premature convergence induced by single update rules, hybrid mutation strategies are introduced through phased mutation operators and elite dimension inheritance mechanisms, effectively reducing premature convergence risks. Experiments demonstrate that the ADVCSO significantly outperforms state-of-the-art algorithms on 27 of 29 CEC2017 benchmark functions, achieving a 2–3 orders of magnitude improvement in convergence precision over basic CSO. In complex composite scenarios, its convergence accuracy approaches that of the championship algorithm JADE within a 10^−2^ magnitude difference. For collaborative multi-subproblem optimization, the ADVCSO exhibits a superior performance in both Multiple Traveling Salesman Problems (MTSPs) and Multiple Knapsack Problems (MKPs), reducing the maximum path length in MTSPs by 6.0% to 358.27 units while enhancing the MKP optimal solution success rate by 62.5%. The proposed algorithm demonstrates an exceptional performance in combinatorial optimization and holds a significant engineering application value.

## 1. Introduction

With the rapid advancement of intelligent manufacturing and IoT technologies, multi-subproblem collaborative optimization scenarios have become prevalent in engineering applications, such as path planning and warehouse scheduling. However, traditional mathematical programming methods often encounter challenges of a high computational complexity and susceptibility to local optima when handling high-dimensional nonlinear and multimodal optimization problems [1]. In recent years, swarm intelligence algorithms inspired by biological collective behaviors have emerged as powerful tools for addressing complex optimization problems due to their self-organizing and parallel search characteristics [2]. Notable examples, including the Snake Optimizer (SO) [3], Divine Religions Algorithm (DRA) [4], Artificial Protozoa Optimizer (APO) [5], Red-Billed Blue Magpie Optimizer (RBMO) [6], Hiking Optimization Algorithms (HOAs) [7], and Sled Dog-Inspired Optimizer (SDO) [8], have demonstrated significant success in engineering optimization and parameter tuning [9]. Consequently, developing efficient and robust novel intelligent optimization algorithms holds substantial theoretical significance and practical value.

The field of swarm intelligence optimization has witnessed a proliferation of innovative bio-inspired algorithms. Representative developments include the Golden Jackal Optimization (GJO) inspired by cooperative hunting behaviors [10], the Butterfly Optimization Algorithm (BOA) based on pheromone diffusion mechanisms [11], and the Sparrow Search Algorithm (SSA) mimicking sparrows’ exploration-following patterns [12]. While these algorithms exhibit distinct advantages across various optimization problems, selecting appropriate algorithms according to specific problem characteristics remains crucial for enhancing solution efficiency.

Despite significant progress in swarm intelligence optimization algorithms for addressing complex optimization challenges, several inherent limitations persist. To tackle these issues, researchers have proposed various innovative improvements. For instance, to address the limitations of the Sand Cat Swarm Optimization (SCSO) algorithm, which suffers from an excessive reliance on the current best solution leading to search stagnation and local optima entrapment, Adegboye et al. integrated Dynamic Pinhole Imaging and the Golden Sine Algorithm, significantly enhancing the algorithm’s global search capability [13]. To overcome the challenge of the insufficient precision in parameter extraction for photovoltaic models using the Hippopotamus Optimization (HO) algorithm, Wang et al. introduced Lévy flight strategies, quadratic interpolation mechanisms, and a swarm elite learning mechanism, markedly improving the algorithm’s solution accuracy and robustness, thereby providing high-quality parameter configurations for photovoltaic systems [14]. For the Dung Beetle Optimizer (DBO), whose performance is unstable due to parameter sensitivity, Xia et al. proposed adaptive dynamic parameter adjustment strategies and a linear scaling method to fine-tune individual positions within dynamic boundaries, effectively maintaining the population diversity while boosting algorithmic stability [15]. For the Parrot Optimizer (PO) algorithm, which struggles with imbalanced exploration-exploitation dynamics, Adegboye et al. incorporated Competitive Swarm Optimization (CSO) and the Salp Swarm Algorithm (SSA) into the PO framework, thereby strengthening the solution diversity and exploratory behavior [16]. These cutting-edge advancements provide novel methodologies for solving real-world complex optimization problems.

The Chicken Swarm Optimization (CSO) algorithm, proposed by Meng et al. in 2014 [17], has gained widespread adoption in resource scheduling and path optimization due to its clear structure and ease of implementation. However, no single algorithm is universally applicable to all optimization problems, prompting researchers to develop CSO enhancements. To address CSO’s premature convergence issue, Liang et al. introduced replication and elimination–dispersal operations from the bacterial foraging algorithm (BFA), replacing weak chicks with poor optimization capabilities, and integrated the collective collaboration mechanism of particle swarm optimization (PSO) to enhance the global search efficiency through subgroup division and information exchange [18]. However, the algorithm parameters require manual configuration, and its significantly increased computational complexity renders it unsuitable for large-scale optimization problems. To improve CSO’s ability to escape local optima in charger placement problems and enhance population utilization, Sanchari et al. incorporated the random walk mechanism of the Ant Lion Optimization (ALO) algorithm, boosting global exploration via a multi-strategy collaboration approach [19]. Nevertheless, limitations remain, including initialization strategies disconnected from solution space characteristics and rigid role allocation mechanisms. To overcome CSO’s inherent weaknesses in solution precision and convergence speed, Li et al. proposed an X-best-guided strategy combined with Lévy flight step sizes for rooster position updates, alongside a dynamic constraint mechanism to maintain population diversity [20]. However, the oversimplified diversity preservation strategy still leads to population homogenization over prolonged iterations due to the elite individual dominance. Addressing CSO’s inefficiency in locating faulty nodes within wireless sensor networks and its inability to distinguish between software and hardware failure types, Nagarajan et al. proposed dynamically adjusting subgroup numbers using fuzzy rules and integrated a Poisson Hidden Markov Model for probabilistic modeling [21]. While this approach optimizes search efficiency and enhances detection accuracy, its parameter settings rely heavily on prior experience, lacking adaptive adjustment capabilities. To tackle CSO’s slow convergence and poor adaptability to dynamic environments, which hinder its application in battery state-of-charge estimations, Afzal et al. incorporated open-ended learning with a dynamic knowledge update mechanism [22]. This enables real-time population updates and historical best-solution retention, effectively improving the algorithm robustness. However, the significantly increased complexity limits its practical deployment. Aiming to resolve CSO’s susceptibility to local optima and the trade-off between convergence speed and precision in complex problems, Gamal et al. developed a hybrid Chicken Swarm Genetic Algorithm (GSOGA) [23]. While CSO accelerates the rapid search and convergence, GA enhances the population diversity through crossover and mutation operations, mitigating local stagnation. Though this hybrid method improves the overall performance, it incurs substantial computational overhead when handling large-scale problems. In contrast, Wang et al. proposed an Adaptive Fuzzy Chicken Swarm Optimization (FCSO) [24], embedding fuzzy logic to monitor optimization velocity and population density during iterations. This self-adjusting mechanism balances exploration and exploitation, while a cosine function integration into the rooster position updates boosts the convergence speed and local refinement. However, the algorithm exhibits limited adaptability in high-dimensional complex optimization scenarios. For multi-objective optimization challenges where CSO struggles with search efficiency and solution quality, Huang et al. introduced a Non-Dominated Sorting Chicken Swarm Algorithm (NSCSO) [25]. By incorporating fast non-dominated sorting, elite opposition-based learning, and a crowding distance strategy, the method enhances the solution diversity and distribution. Despite these advancements, the NSCSO underperforms on highly nonlinear problems and remains overly dependent on the quality of initial randomized population positions. Table 1 summarizes these CSO improvement efforts.

In conclusion, while CSO outperforms many swarm intelligence algorithms and has inspired numerous enhanced variants, it still grapples with inherent limitations such as low solution precision, sluggish convergence rates, and premature stagnation. This study proposes an Adaptive Dynamically Enhanced Variant of Chicken Swarm Optimization (ADVCSO) through three key innovations. First, an elite perturbation initialization strategy based on good point sets generates uniformly distributed initial populations using low-discrepancy sequences, enhanced by the Gaussian perturbation of elite individuals for local exploration. Second, a population diversity-based dynamic role allocation mechanism adaptively adjusts subgroup proportions and update cycles through cosine annealing scheduling, effectively balancing exploration and exploitation. Third, a hybrid mutation strategy incorporating Cauchy–Gaussian phased mutation operators and an elite dimension inheritance enables a dynamic equilibrium between global exploration and local refinement. These synergistic enhancements significantly improve the population diversity maintenance, adaptive role allocation, and local optima avoidance, establishing a novel methodological framework for multi-subproblem collaborative optimization.

The principal contributions of this work include the following:

1. The development of an elite perturbation initialization strategy based on good point sets, which not only significantly enhances the quality of initial solutions but also improves the population’s overall search capability;
2. Designed a dynamic role allocation mechanism based on population diversity, effectively maintaining population diversity and strengthening the algorithm’s self-adaptive capabilities;
3. Implemented a hybrid mutation strategy for population position updates, incorporating Cauchy–Gaussian phased mutation operators and an elite dimension inheritance strategy to bolster the algorithm’s ability to escape local optima and effectively mitigate premature convergence risks;
4. Validated through ablation studies, the CEC2017 benchmark functions, Multi-Traveling Salesman Problems, and Multi-Knapsack Problems, the ADVCSO demonstrates an exceptional global search capability and stability, offering a novel paradigm for practical combinatorial optimization challenges.

The remainder of this paper is organized as follows: Section 2 outlines the fundamental principles of CSO. Section 3 details the ADVCSO methodology and enhancement strategies. Section 4 presents ablation studies on the ADVCSO, followed by experiments on the CEC2017 test function suite and applications to two real-world combinatorial optimization problems. Finally, Section 5 concludes with discussions on algorithmic innovations, experimental findings, limitations, and future research directions.

## 2. Chicken Swarm Optimization Algorithm

The Chicken Swarm Optimization (CSO) algorithm simulates the hierarchical structure and foraging behaviors of chicken societies and abstracts them into an optimization model. In this framework, each individual in the population represents a potential solution, classified into roles—roosters, hens, and chicks—based on their fitness values, and the flock is divided into several subgroups corresponding to the number of roosters. Specifically, roosters are the optimal individuals leading each subgroup, and chicks are those with the poorest fitness and lowest status, while the remaining individuals are designated as hens, with some randomly selected as mother hens with assigned chicks. During foraging, roosters lead their subgroups in search of food, hens follow roosters to search for or compete for high-quality food, while chicks can only forage by following their mother hens. Once roles are assigned, they remain fixed until re-evaluated and re-established after G generations of iterations.

In the algorithm, let N denote the total population size, with $N_{R}$, $N_{H}$, and $N_{C}$ representing the numbers of roosters, hens, and chicks, respectively. The position of the i-th individual in the j-th dimension at iteration t is denoted as $x_{i,j}^{t}$. The initial population is generated randomly within the search space using Equation (1):

(1) $$x_{i}=lb+Rand\left(ub-lb\right)$$

where ub and lb represent the upper and lower bounds, and Rand is a random number uniformly distributed in $\left[0,1\right]$.

The individuals with the lowest fitness value are selected as the roosters. As leaders of subgroups, roosters autonomously determine foraging directions. Their position is updated, as shown in Equations (2) and (3):

(2) $$x_{i,j}^{t+1}=x_{i,j}^{t}\cdot \left(1+N\left(0,\sigma^{2}\right)\right)$$

(3) $$\sigma^{2}=\left\{\begin{array}{c} 1,\;if\;f_{i}<f_{k}\\ \exp\frac{\left(f_{k}-f_{i}\right)}{\left|f_{i}\right|+\varepsilon },\;\mathrm{otherwise} \end{array}\right.,k\in \left[1,N_{R}\right],k\neq i$$

where $N\left(0,\sigma^{2}\right)$ is a Gaussian distribution random number. The $f_{i}$ and $f_{k}$ denote the fitness values of the current rooster and a randomly selected rooster, respectively. ε is a small constant to prevent a division by zero.

Individuals with better fitness are selected as hens, which move following their subgroup’s rooster and may scavenge better food resources. The hen’s position is updated, as shown in Equations (4) and (5):

(4) $$x_{i,j}^{t+1}=x_{i,j}^{t}+S_{1}\cdot Rand\cdot \left(x_{r1,j}^{t}-x_{i,j}^{t}\right)+S_{2}\cdot Rand\cdot \left(x_{r2,j}^{t}-x_{i,j}^{t}\right)$$

(5) $$S_{1}=exp\left(\frac{f_{i}-f_{r1}}{\left|f_{i}\right|+\varepsilon }\right),\;\;\;\;\;\;S_{2}=exp\left(f_{r2}-f_{i}\right)$$

where $x_{r1,j}^{t}$ is the position of the hen’s mate rooster, specifically the rooster of the subgroup to which the hen belongs. $x_{r2,j}^{t}$ is a randomly selected rooster or hen (r1 ≠ r2).

Except for the roosters and hens, other individuals are defined as chicks. The chicks strictly follow their mother hens during foraging, and the chick’s position is updated, as shown in Equation (6):

(6) $$x_{i,j}^{t+1}=x_{i,j}^{t}+FL\cdot \left(x_{m,j}^{t}-x_{i,j}^{t}\right)$$

where FL∈[0,2], $x_{m,j}^{t}$ is the position of the chick’s mother hen.

## 3. ADVCSO Algorithm

The original CSO algorithm demonstrates strengths in low-dimensional function optimization through its role-based cooperative mechanism. However, its application to multi-subproblem collaborative optimization faces three critical challenges: (1) an uneven initial solution distribution caused by random initialization, leading to an inadequate search space coverage; (2) rigid role proportions and update cycles that fail to adapt to dynamic requirements across optimization phases, resulting in a significant exploration–exploitation imbalance; and (3) the limited ability of single mutation strategies to escape local optima in complex multimodal problems. To address these challenges, this study proposes the ADVCSO algorithm, which integrates good-point-set-based elite perturbation initialization, a population diversity-driven dynamic role mechanism, and hybrid mutation strategies. This section elaborates on the ADVCSO algorithm, its enhancement strategies, and establishes the ADVCSO mathematical model.

### 3.1. Good-Point-Set-Based Elite Perturbation Initialization Strategy

In natural environments, chicken swarms often exhibit spatial regularity in their distribution rather than complete randomness. However, the original CSO employs random initialization, leading to sparse solutions in certain dimensions and low-quality initial populations. Inspired by biological principles, this study designs a good-point-set-based elite perturbation initialization strategy to replace random initialization, mimicking the natural distribution patterns of chicken swarms.

The good point set can be mathematically described as follows: Let $V_{D}$ denote a D-dimensional unit hypercube in the search space. A set of points, $r\in V_{D}$, is termed a good point set and is defined by Equation (7):

(7) $$P_{n}(k)=\left\{\left(\left\{r_{1}^{\left(n\right)}*k\right\},\left\{r_{2}^{\left(n\right)}*k\right\},\ldots ,\left\{r_{D}^{\left(n\right)}*k\right\}\right),1\leq k\leq n\right\},$$

where r represents the good point, $\left\{r_{D}^{\left(n\right)}*k\right\}$ denotes the decimal part within it, and n is the number of good points. A key characteristic of good point sets is their extremely small discrepancy, representing a distribution that approaches complete uniformity. The magnitude of individual discrepancies $\varphi_{n}$ is measured by the deviation, which satisfies Equation (8):

(8) $$\varphi_{n}=C(r,\varepsilon )n^{-1+\varepsilon },$$

where C(r,ε) is a constant dependent on r and ε (ε is an arbitrary positive integer). Equation (8) demonstrates that as the number of points increases, the discrepancy will decrease [26].

To generate the good point set, this study adopts the cosine sequence method to determine the parameter r. The calculation is defined by Equation (9):

(9) $$r=\left\{2\cos\left(2\pi k/p\right),1\leq k\leq D\right\}$$

where p is the smallest prime number satisfying $\left(p-3\right)/2\geq D$.

Furthermore, to simulate the localized exploration behavior of leading roosters in chicken swarms, a small-scale Gaussian perturbation is applied to the top 10% of elite individuals (by fitness). The perturbation is formulated in Equation (10):

(10) $$x_{i,j}^{t+1}=x_{i,j}^{t}+\eta \cdot \left(ub-lb\right)\cdot N\left(0,I\right)$$

where η is the perturbation intensity coefficient (set to 0.05 in this study). The perturbation range is dynamically scaled by $\left(ub-lb\right)$ to align with the search space dimensions.

**Figure 1:**
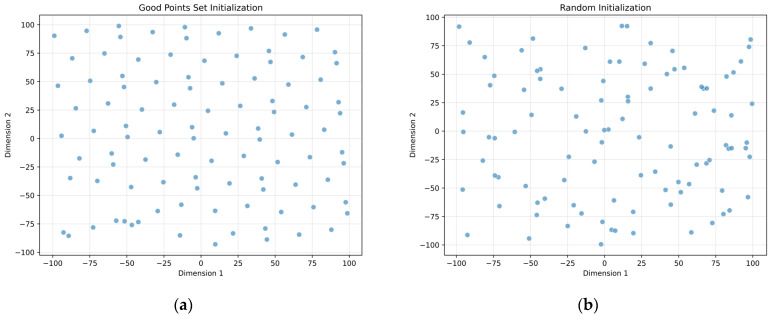
Initialization performance comparison. (**a**) Good-point-set-based elite perturbation initialization. (**b**) Random initialization.

Figure 1 compares population initialization results using the good point set versus random initialization. It clearly demonstrates that the good point set method generates an initial population that more uniformly covers the search space, while the Gaussian perturbation prevents clustering in localized regions.

### 3.2. Population Diversity-Driven Dynamic Role Allocation Mechanism

In natural environments, the role proportions within chicken swarms dynamically adapt to resource availability: during resource abundance, the ratios of hens and chicks increase to promote population reproduction, whereas under resource scarcity or threats, rooster proportions rise to enhance the collective exploration and defense capabilities. However, the original CSO employs fixed role proportions, failing to adapt to varying demands across optimization phases. Inspired by this biological behavior, this study proposes a population diversity-driven dynamic role allocation mechanism. By the real-time monitoring of population diversity states, the mechanism dynamically adjusts role proportions: increasing hen and chick ratios to drive local refinement when diversity is high and expanding rooster proportions to strengthen global exploration when diversity declines significantly. This adaptive mechanism shifts the search focus based on the optimization progress, significantly enhancing the algorithm robustness in complex multimodal problems. The specific calculation process is as follows:

The population diversity metric is defined as the standard deviation of fitness values, calculated via Equation (11):

(11) $$\sigma_{t}=\sqrt{\frac{1}{N}\sum_{i=1}^{N}{\left(f_{i}-\bar{f}\right)}^{2}}$$

where $\sigma_{t}$ represents population diversity at iteration t. $\bar{f}=\frac{1}{N}\sum_{i=1}^{N}f_{i}$ denotes the average population fitness.

A diversity decay ratio ρ is introduced to dynamically regulate role proportions. The updated role allocation formulas are defined in Equations (12)–(15):

(12) $$\rho =\frac{\sigma_{0}}{\sigma_{t}}$$

(13) $$N_{R}=max(1,min(N\cdot (0.3+0.2\rho ),N-2))$$

(14) $$N_{H}=max(1,min(N\cdot (0.3-0.1\rho ),\;\;\;\;N-N_{R}-1))$$

(15) $$N_{C}=max(0,N-N_{R}-N_{H})$$

**Figure 2:**
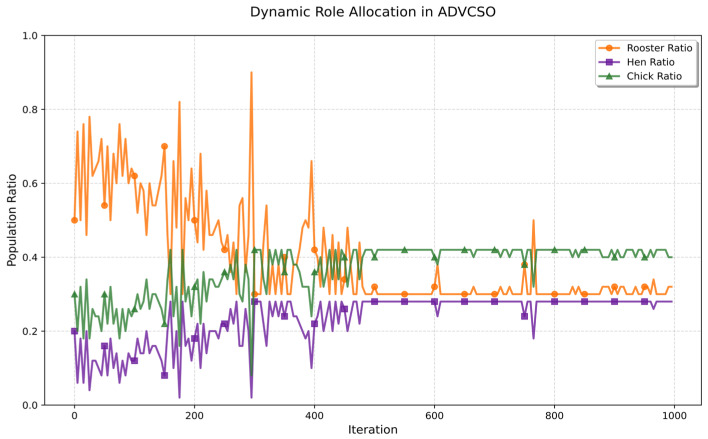
The effect of the dynamic role allocation mechanism.

As shown in Figure 2, the ADVCSO algorithm exhibits significant dynamic changes in role proportions throughout the iteration process. In the early iterations, the rooster proportion fluctuates frequently and remains generally high, with peaks exceeding 80%, indicating that the algorithm is in the exploration phase, strengthening the global search by increasing the proportion of roosters; correspondingly, the hen proportion decreases, reducing the resource allocation for local exploitation. As iterations progress, fluctuations in role proportions gradually diminish, with the rooster proportion decreasing to approximately 30%, while hen and chick proportions stabilize at around 28% and 42%, respectively, reflecting a balance between exploration and exploitation. In the later stages of the algorithm iteration, the rooster proportion continues to exhibit small fluctuations, demonstrating that the algorithm maintains certain exploration capabilities to prevent premature convergence. Notably, the rooster proportion shows sudden increases near iterations 300, 390, and 750, which aligns with the algorithm’s need to continuously escape from local optima traps. This diversity-aware dynamic role allocation mechanism adjusts the search focus according to the optimization progress, significantly enhancing the algorithm’s robustness in complex multimodal problems.

Additionally, ADVCSO incorporates a cosine annealing strategy to dynamically adjust the role update cycle G. A nonlinear decay function progressively compresses G, maintaining a larger G in early iterations for a thorough exploration and reducing G in later stages to accelerate local convergence. This approach mitigates the oscillation caused by frequent role switching while balancing the convergence speed and precision. The update rules are formulated in Equations (16) and (17):

(16) $$\mathrm{decay}\left(t\right)=0.5\;(1+cos(\frac{\pi t}{T}))$$

(17) $$G_{current}=max(5,(G\cdot decay\left(t\right)))$$

### 3.3. Hybrid Mutation Strategy

Chicken swarms exhibit complex social learning mechanisms, where individuals of different hierarchies demonstrate distinct behavioral traits. However, the original CSO employs a single position update strategy, rendering it prone to local optima, particularly in complex multimodal problems. Inspired by biological principles, this study proposes a hybrid mutation strategy to address these limitations.

#### 3.3.1. Rooster Mutation Strategy Based on Behavioral Differences

In nature, roosters’ foraging behaviors exhibit distinct phases: large-scale exploratory leaps when exploring new territories and small-scale refined searches within familiar areas. This behavioral dichotomy motivates the design of a Cauchy–Gaussian phased mutation strategy. The Cauchy distribution, with its heavy-tailed properties, facilitates long-range jumps in the search space to enhance the global exploration, while the Gaussian distribution introduces minor stochastic variations for localized refinement. To optimize the ADVCSO’s search efficiency, the mutation mode is adaptively switched based on optimization phases: the Cauchy mutation is applied when population diversity is low, and the Gaussian mutation is activated as diversity improves. The mutation-enhanced position update is governed by Equations (18)–(20):

(18) $$\tau \left(t\right)=0.3+0.4\left(1-\rho \right)$$

(19) $$\xi \sim \left\{\begin{array}{c} \mathrm{Cauchy}\left(0,1\right),\;t<\tau \left(t\right)\cdot T\\ 0.5\cdot N\left(0,1\right),\;\mathrm{otherwise} \end{array}\right.$$

(20) $$x_{i,j}^{t+1}=x_{i,j}^{t}+\alpha \cdot \left(ub-lb\right)\cdot \xi$$

where $\tau \left(t\right)$ is the mutation switching threshold, controlling the transition between Cauchy and Gaussian mutations. α is the mutation intensity coefficient (empirically set to 0.05 to avoid elite individual loss).

Additionally, inspired by roosters’ age-dependent behavioral shifts—younger roosters prioritize exploration, while experienced ones focus on territorial defense—an adaptive learning factor $\alpha \left(t\right)$ is introduced to modulate position updates. This adaptive learning factor $\alpha \left(t\right)$ simulates how roosters adjust their behavioral patterns as they gain experience. In the early iterations, when t is small, the learning factor $\alpha \left(t\right)$ is also small, representing young roosters leading their subgroups in a global search; as the number of iterations increases, roosters gain experience, their behavior shifts toward refined exploitation, and the learning factor $\alpha \left(t\right)$ increases accordingly, further regulating the roosters’ position updates. The learning factor evolves nonlinearly with iterations, as formulated in Equations (21) and (22):

(21) $$\alpha \left(t\right)=0.5\left(1-cos\left(\frac{\pi t}{T}\right)\right)$$

(22) $$x_{i,j}^{t+1}=x_{i,j}^{t}\cdot \left(1+\alpha \left(t\right)\cdot N\left(0,\sigma^{2}\right)\right)$$

#### 3.3.2. Dimension Updating and Elite Inheritance Mechanism Based on Social Learning

In chicken societies, hens and chicks learn successful behaviors from roosters through observation and imitation, particularly in foraging and risk avoidance. However, the original CSO suffers from inefficiency: hens randomly learn from any individual, while chicks rigidly follow their mother hens, risking local optima entrapment. To address this, a dimension learning and elite inheritance mechanism is proposed. This mechanism mimics selective learning from leader roosters while preserving individuality through minor perturbations, effectively balancing exploitation efficiency and diversity maintenance. The specific calculation process is as follows:

During position updates, hens probabilistically learn specific dimensions from the global best solution (leader rooster). The update is defined by Equations (23) and (24):

(23) $$D\subseteq \left\{1,2,\ldots ,D\right\},\left|D\right|=\left\lfloor 0.2D\right\rfloor$$

(24) $$x_{i,d}^{t+1}=\left\{\begin{array}{c} x_{g,d}^{t^{*}}+\beta \cdot \left({ub}_{d}-{lb}_{d}\right)\cdot N\left(0,1\right),\;d\in D\\ x_{i,d}^{t},\;\mathrm{otherwise} \end{array}\right.$$

where D is a randomly selected subset of dimensions. β is the learning intensity coefficient (set to 0.05). $\left({ub}_{d}-{lb}_{d}\right)$ are the dynamic scale perturbations.

Chicks probabilistically inherit critical dimensions from the global best solution. The update follows Equations (25) and (26):

(25) $$D_{elite}\subseteq \left\{1,2,\ldots ,D\right\},\;\;\left|D_{elite}\right|=2$$

(26) $$x_{i,d}^{t+1}=\left\{\begin{array}{c} x_{g,d}^{t^{*}},\;\mathrm{if}\;\mathrm{Rand}<\omega ,\;d\in D_{elite}\\ x_{i,d},\;\mathrm{otherwise} \end{array}\right.$$

where $D_{elite}$ denotes two randomly selected elite dimensions. ω is the inheritance probability (set to 0.2).

Additionally, in chicken swarms, the following behavior of hens is influenced by their social status within the group. High-ranking hens tend to forage independently, while low-ranking hens rely heavily on following roosters. However, the original CSO neglects individual status differences, resulting in an over-reliance on random exploration by low-fitness individuals. To address this, this study introduces a rank-adaptive strategy to modulate hens’ following behaviors, with specific update rules defined in Equations (27)–(29):

(27) $$\gamma_{i}=1-\frac{rank\left(i\right)}{N},\;rank\left(i\right)\in \left\{1,2,\ldots ,N\right\}$$

(28) $$S_{1}=exp\left(\frac{f_{i}-f_{r1}}{\left|f_{i}\right|+\varepsilon }\right),\;S_{2}=\gamma_{i}\cdot exp\left(f_{r2}-f_{i}\right)$$

(29) $$x_{i,j}^{t+1}=x_{i,j}^{t}+S_{1}\cdot Rand\cdot \left(x_{r1,j}^{t}-x_{i,j}^{t}\right)+S_{2}\cdot Rand\cdot \left(x_{r2,j}^{t}-x_{i,j}^{t}\right)$$

where $\gamma_{i}$ represents the fitness-based rank of individual i within the population.

### 3.4. Architecture of ADVCSO

**Figure 3:**
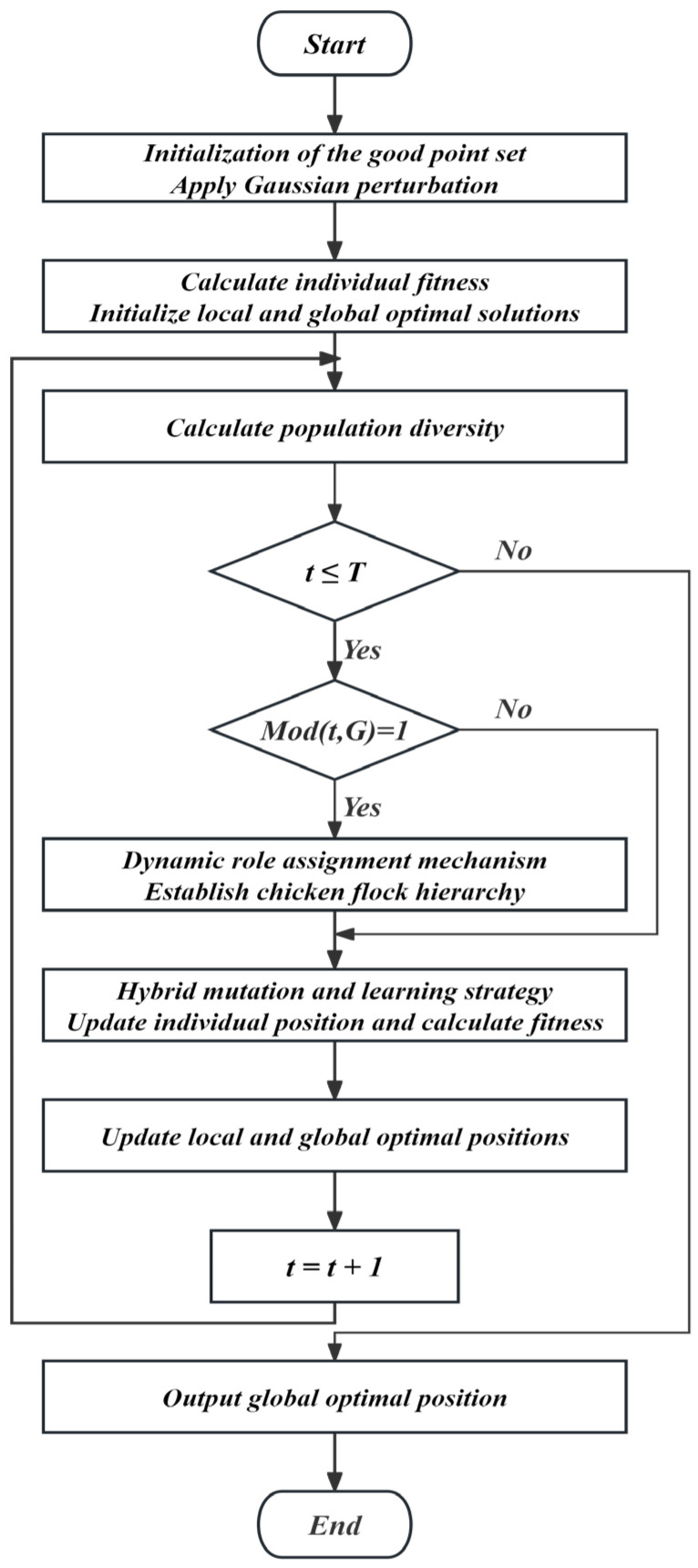
The flowchart of the ADVCSO algorithm.

To address the premature convergence issue encountered by the basic CSO algorithm in solving high-dimensional optimization problems, the ADVCSO algorithm is proposed. The algorithm integrates three key enhancement strategies into a cohesive framework: the good point set initialization, population diversity-driven dynamic role allocation, and hybrid mutation strategy. Algorithm 1 provides the pseudocode implementation, detailing the specific steps and processes. The overall architecture of the ADVCSO is illustrated in Figure 3, which illustrates the algorithmic flowchart of the algorithm.
**Algorithm 1:** Pseudocode of ADVCSO algorithm**Initialize** population using good point set and apply perturbation to top 10% of elites;**Define** parameters: epoch, G_max_, pop_size, N, G, and jixiaoliang;1:**While** t < G_max_:2:    **If** (t % G_current_ == 0):3:        Sort population by fitness and dynamically assign roles:4:           -Roosters (N_R_): top-ranked individuals5:           -Hens (N_H_): middle-ranked individuals6:           -Chicks (N_C_): remaining individuals7:        Update mating pairs and mother–chick relationships;8:        Adaptively adjust G_current_ via cosine annealing;9:
    **End if**
10:    **For** i = 1 to N_R_:11:        Update roosters’ positions with adaptive learning factor;12:        Apply hybrid mutation (Cauchy/Gaussian based on diversity threshold);13:
    **End for**
14:    **For** i = 1 to N_H_:15:        Update hens’ positions using rank-based following strategy;16:        Replace partial dimensions with global best values;17:
    **End for**
18:    **For** i = 1 to N_C_:19:        Update chicks’ positions via spiral learning with FL factor;20:        Inherit elite dimensions from global best;21:
    **End for**
22:    Evaluate new solutions and update local/global best;23:    Adjust mutation threshold based on population diversity;24:**End while**25:**Return** global best solution;

1. Initialization Module: The algorithm initializes the population using a good point set based on low-discrepancy sequences, which generates uniformly distributed initial solutions across the search space. This strategy achieves the following:
  (a) Creates an initial population where individuals follow a quasi-random distribution rather than complete randomness.
  (b) Applies perturbation to the top 10% elite individuals to avoid early stagnation.
  (c) Enhances the quality and diversity of initial solutions, significantly improving the search space coverage.
2. Role Allocation Manager: based on population diversity metrics, this module dynamically assigns roles (roosters, hens, and chicks) using the following process:
  (a) The population diversity calculation according to Equation (11).
  (b) The dynamic adjustment of role proportions following Equations (12)–(15).
  (c) Sorting individuals by fitness and assigning roles based on calculated proportions.
  (d) The establishment of hierarchical relationships including mate pairs and mother–chick relationships.
3. Hybrid Mutation Strategy: this module implements an adaptive mutation and dimension learning mechanism that adheres to the following process:
  (a) Switches between the Cauchy distribution for global exploration and the Gaussian distribution for local refinement based on the search stage and diversity threshold defined in Equation (18).
  (b) Incorporates an adaptive learning factor that evolves nonlinearly with iterations according to Equations (21) and (22).
  (c) Applies a selective mutation based on the individual status and population diversity state.
  (d) Enables hens to learn specific dimensions from the global best solution with a probability of 0.2.
  (e) Allows chicks to inherit elite dimensions from global best solutions with a controlled probability.

As shown in Algorithm 1, the ADVCSO implementation begins with the initialization of key parameters, followed by population generation using the good point set method. The dynamic role allocation mechanism is executed at lines 3–7 of the algorithm, where the population diversity is calculated and role proportions are adjusted accordingly. Lines 10–21 detail the position update mechanism for different roles, incorporating the hybrid mutation strategy described in Section 3.3.

The interactions between these architectural components follow the precise workflow illustrated in Figure 3:

1. The population diversity is measured;
2. Role proportions are adjusted according to the diversity ratio;
3. Roles are assigned based on fitness rankings;
4. Position updating occurs using role-specific rules enhanced with the hybrid mutation;
5. The mutation mode switches adaptively based on the population state and iteration progress;
6. Dimension learning enables effective knowledge transfer between solutions.

This modular design effectively balances exploration and exploitation capabilities, significantly enhancing the algorithm robustness in complex multimodal problems. The comprehensive architecture not only addresses the limitations of the original CSO—including the initial distribution quality, rigid role proportions, and single mutation strategy—but also creates a synergistic effect through the systematic integration of these enhancement strategies, as evidenced by the performance improvements demonstrated in the experimental results section.

### 3.5. The Complexity Analysis of the ADVCSO Algorithm

The computational efficiency of metaheuristic algorithms directly impacts their practical applicability to complex optimization problems. This section analyzes the time and space complexity of the proposed ADVCSO algorithm.

#### 3.5.1. Time Complexity

For the time complexity analysis, let N denote the population size, d the problem dimension, T the total number of iterations, and G the update frequency of the role assignment. The time complexity of the ADVCSO consists of initialization and iteration phases.

The initialization phase includes parameter setting, population generation using good point sets, and an initial fitness evaluation. The good point set initialization requires generating appropriate prime numbers ($O(\sqrt{d\cdot \log d})$) and creating low-discrepancy sequences for each individual (O(N·d)). Combined with the initial fitness evaluation ($O(N\cdot f_{e})$, where $f_{e}$ is the cost of a single fitness evaluation), the total initialization complexity is $O(N\cdot d+N\cdot f_{e})$.

During the iteration phase, each iteration involves several key operations. The population diversity calculation and role assignment require O(N·log⁡N) operations but are only performed every G iterations, contributing $O(T\cdot N\cdot \log\frac{N}{G})$ to the total complexity. The position updating differs for each role (roosters, hens, and chicks), but collectively requires O(N·d) operations per iteration. The hybrid mutation strategy, including dimension learning mechanisms, adds operations that scale with O(N·d). Each iteration also requires a fitness evaluation of the entire population ($O(N\cdot f_{e})$). Considering all these components, the time complexity of the iteration phase is $O(T\cdot N\cdot (d+f_{e}+\log\frac{N}{G}))$. The overall complexity of the ADVCSO is shown in Equation (30):

(30) $$O(ADVCSO)=O(N\cdot d+N\cdot f_{e})+O(T\cdot N\cdot (d+f_{e}+\log\frac{N}{G}))$$

Since the initialization is performed only once, the overall time complexity of the ADVCSO is dominated by the iteration phase: $O(T\cdot N\cdot (d+f_{e}+\log\frac{N}{G}))$. For high-dimensional problems where $d\gg \log\frac{N}{G}$, and considering that $f_{e}$ is often proportional to d, O(ADVCSO) can be approximated by Equation (31):

(31) O(ADVCSO)=O(T·N·d)

In the original CSO, the initialization phase requires O(N·d) operations for random population generation and $O(N\cdot f_{e})$ for fitness evaluation. The iteration phase involves role assignments every G iterations ($O(N\cdot \log\frac{N}{G})$) and position updating for all individuals (O(N·d)). Thus, the total computational complexity of the standard CSO algorithm can be expressed as O(CSO)=O(T·N·d).

This analysis reveals that despite its enhanced features, the ADVCSO maintains the same asymptotic time complexity as the original CSO algorithm.

#### 3.5.2. Space Complexity

The space complexity of the ADVCSO is primarily determined by the storage requirements for the population (O(N·d)), fitness values (O(N)), role assignments (O(N)), and best solutions (O(N·d)). The dominant term is O(N·d), which represents the overall space complexity of the algorithm. Similarly, O(N·d) is the dominant term in CSO’s space complexity. Therefore, both algorithms have the same asymptotic space complexity.

Experimental measurements confirm this theoretical analysis. When tested on CEC2017 benchmark functions with d=30 and N=50, the ADVCSO requires approximately 69.6 ms per iteration, which is comparable to CSO’s 57.7 ms. This slight difference in execution time is negligible considering the significant performance improvements achieved.

This analysis demonstrates that the ADVCSO achieves an enhanced optimization performance without incurring significant additional computational costs, making it suitable for practical applications in high-dimensional and complex optimization scenarios.

## 4. Experimental Design and Analysis

To evaluate the performance of the ADVCSO, ablation studies were first designed to validate the effectiveness of each enhancement strategy, followed by comparative experiments which were conducted against other swarm intelligence algorithms on the CEC2017 benchmark functions, the Multi-Traveling Salesman Problem (MTSP), and the Multi-Knapsack Problem (MKP). The experimental setup included Windows 11 OS, Intel i7-12700H processor, 16.0GB RAM, and Python 3.12.

### 4.1. Ablation Studies

To verify the effectiveness of each enhancement strategy in the ADVCSO algorithm, this study designed a series of ablation experiments by incrementally adding improvement strategies to the original CSO algorithm to analyze each strategy’s contribution to the algorithm performance. The specific algorithm variants are shown in Table 2.

**Figure 4:**
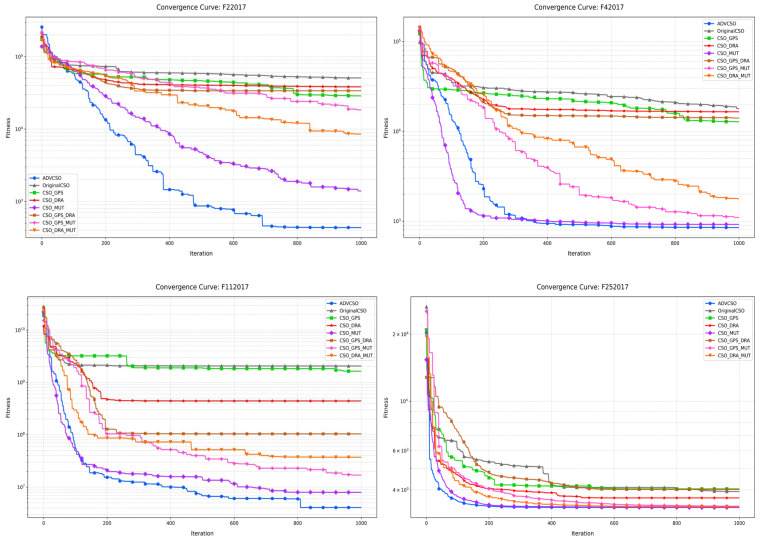
Convergence curve comparison of CSO algorithm variants.

The experiments were conducted on four typical functions from the CEC2017 test suite: unimodal function F2 (F22017), multimodal function F4 (F42017), hybrid function F11 (F112017), and composition function F25 (F252017). Each algorithm variant was run for 1000 iterations on each function, resulting in the convergence curve comparison shown in Figure 4.

#### 4.1.1. Analysis of Individual Enhancement Strategies

The good point set initialization strategy significantly improved the early convergence speed. For example, on F2, CSO_GPS demonstrated a noticeably faster convergence than the original CSO throughout the iteration process, ultimately achieving a fitness value of 2.87 × 10^4^, significantly outperforming the original CSO’s 5.09 × 10^4^. This result indicates that the good point set initialization effectively improved the initial distribution quality of the population, providing better initial solutions and accelerating the early convergence. The improvement was particularly significant on F25, confirming that the good point set initialization enhanced the search space coverage and provided a more uniform initial solution distribution.

The dynamic role allocation mechanism balanced exploration and exploitation capabilities. CSO_DRA exhibited a better performance than the original CSO on most test functions. On function F25, CSO_DRA continued to show an optimization capability after approximately 400 iterations, while the original CSO tended to stabilize, indicating that the dynamic role allocation strategy effectively delayed premature convergence. Notably, on function F4, CSO_DRA’s convergence curve exhibited periodic declining characteristics, directly related to its mechanism of dynamically adjusting role proportions and update cycles. This demonstrates that the strategy can adaptively balance global exploration and local exploitation capabilities at different optimization stages, effectively breaking through search plateaus.

Among all four types of test functions, CSO_MUT showed the best single-strategy improvement effect. On function F2, CSO_MUT achieved a fitness value of 1.40 × 10^3^, nearly an order of magnitude lower than the original CSO. On function F11, CSO_MUT decreased almost synchronously with the ADVCSO, reaching a final fitness value of 7.91 × 10^6^, far lower than other single-strategy variants. Especially on highly nonlinear functions like F25, CSO_MUT’s convergence curve showed multiple breakthrough declines, which were closely related to its phased mutation operator design and elite dimension inheritance mechanism, indicating that this strategy can effectively overcome premature convergence problems caused by single update rules.

#### 4.1.2. Analysis of Synergistic Effects of Combined Strategies

CSO_GPS_DRA showed a better performance than either individual strategy on F2 and F11, indicating a positive synergistic effect between these two strategies. Particularly on F11, CSO_GPS_DRA’s final fitness value was 1.03 × 10^8^, which was significantly lower than CSO_GPS (1.64 × 10^9^) and CSO_DRA (4.40 × 10^8^). This synergistic effect stems from the good point set initialization providing higher quality initial solution distributions, while the dynamic role allocation mechanism more effectively allocates computational resources based on these good initial solutions, further improving search efficiency. However, on F4, CSO_GPS_DRA’s performance improvement was not significant, and even slightly inferior to CSO_DRA at certain iteration stages, suggesting that the combination of these two strategies may have complex interactions on different problem types and does not always produce additive gains.

CSO_GPS_MUT demonstrated an outstanding performance on all test functions. On F4, CSO_GPS_MUT’s convergence curve approached that of the complete ADVCSO, with a final fitness value of approximately 1.10 × 10^3^. On F25, CSO_GPS_MUT performed similarly to CSO_MUT but converged faster in early iteration stages, proving that the good point set initialization and hybrid mutation strategy have significant complementarity: the good point set initialization provides high-quality initial solutions, offering a better mutation foundation for the hybrid mutation strategy, thereby improving the algorithm’s search efficiency.

CSO_DRA_MUT exhibited the best overall performance among all dual-strategy combinations. On F2, CSO_DRA_MUT achieved a final fitness value of 8.53 × 10^3^, performing the best among all dual-strategy combinations. Its periodic breakthrough convergence characteristics were particularly evident, indicating that the dynamic role allocation ensures the efficient allocation of computational resources, while the hybrid mutation strategy provides the ability to escape local optima, creating a powerful synergistic effect when combined.

#### 4.1.3. The Performance Analysis of the Complete ADVCSO

The ADVCSO, integrating all three improvement strategies, demonstrated the best performance on all four function types. Taking F2 as an example, the ADVCSO achieved a fitness value of 4.35 × 10^2^, which is an order of magnitude lower than the best dual-strategy combination CSO_DRA_MUT (8.53 × 10^3^) and nearly two orders of magnitude lower than the original CSO (5.09 × 10^4^). During the convergence process, the ADVCSO’s convergence curve exhibited typical “staircase” descent characteristics, indicating the algorithm’s ability to repeatedly break through local optima and continuously find better solutions. This characteristic stems from the synergistic effect of the three improvement strategies: the good point set initialization provides high-quality initial solutions, the dynamic role allocation ensures efficient resource allocation, and the hybrid mutation strategy provides the ability to escape local optima.

The results of the ablation experiments indicate that among the three improvement strategies, the hybrid mutation strategy contributed most significantly to the algorithm performance, followed by the dynamic role allocation strategy, while the good point set initialization strategy showed notable effects in the early convergence stage. Different strategy combinations typically produced synergistic effects, with the combination of the dynamic role allocation and hybrid mutation being particularly outstanding, complementing each other’s advantages and jointly improving the algorithm’s exploration–exploitation balance. After integrating all improvement strategies, the complete ADVCSO algorithm formed a more powerful synergistic effect among strategies, enabling the algorithm to demonstrate an excellent performance when addressing different types of optimization problems, thus validating the effectiveness of the improvement methods proposed in this study.

### 4.2. CEC2017 Benchmark Experiments

In this study, Table 3 is utilized to evaluate the performance of the ADVCSO, which includes 29 test functions from the CEC2017 benchmark suite. These functions exhibit diverse characteristics, covering unimodal, multimodal, hybrid, and composite types, designed to assess algorithmic capabilities in local exploitation, global exploration, and other critical performance metrics [27] (note that the original F2 function in the CEC2017 test suite was officially removed due to defects). In addition to comparisons with the original CSO, this study selects several state-of-the-art algorithms that have demonstrated outstanding performances in recent IEEE CEC competitions for benchmarking: the Giant Trevally Optimizer (GTO) [28], Coyote Optimization Algorithm (COA) [29], Hunger Games Search (HGS) [30], and Sea Lion Optimization Algorithm (SLO) [31]. To ensure the reliability of the test results, all algorithms are uniformly configured with a problem dimensionality of 30, a population size of 50, and 1000 iterations. Table 4 lists the key initial parameter settings for each algorithm. These parameter selections are informed by a comprehensive understanding of the problem domain and insights from prior research, aiming to enhance the algorithms’ effectiveness and generalizability across diverse datasets and scenarios.

**Figure 5:**
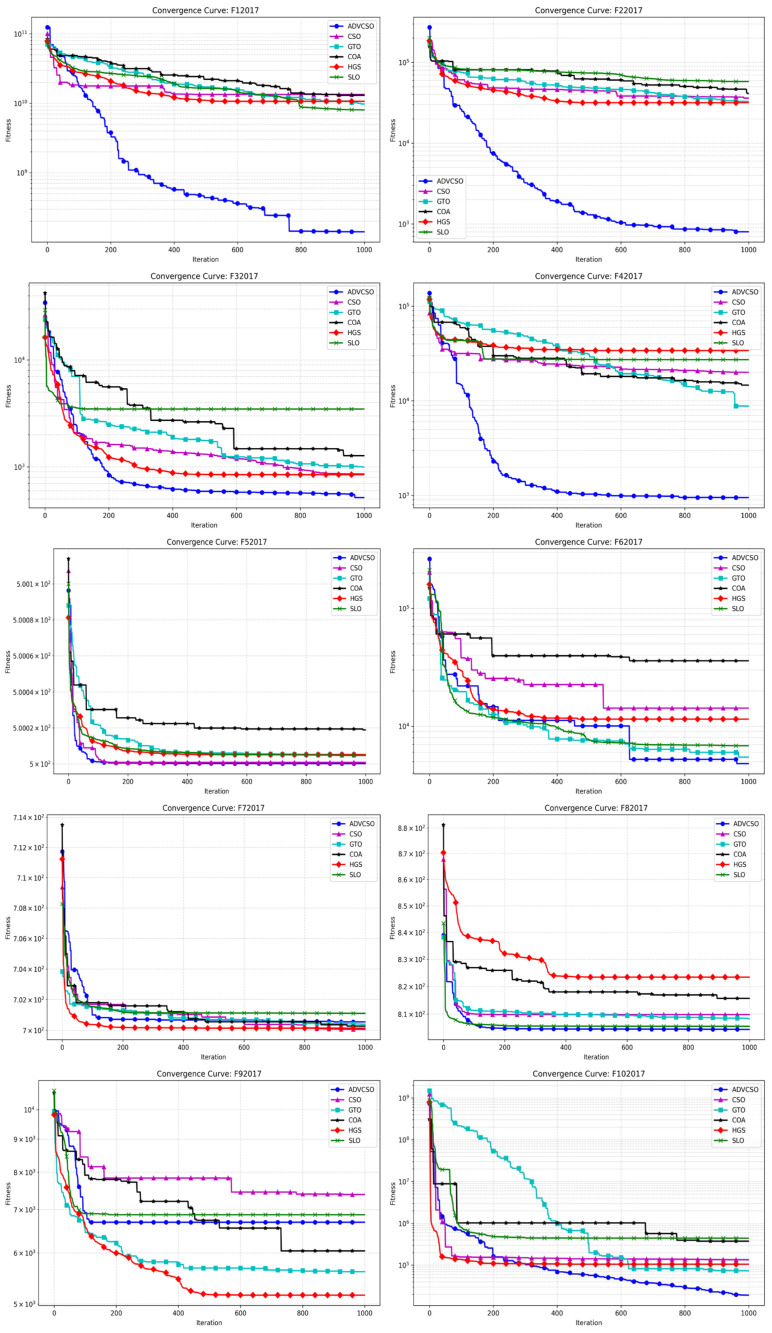

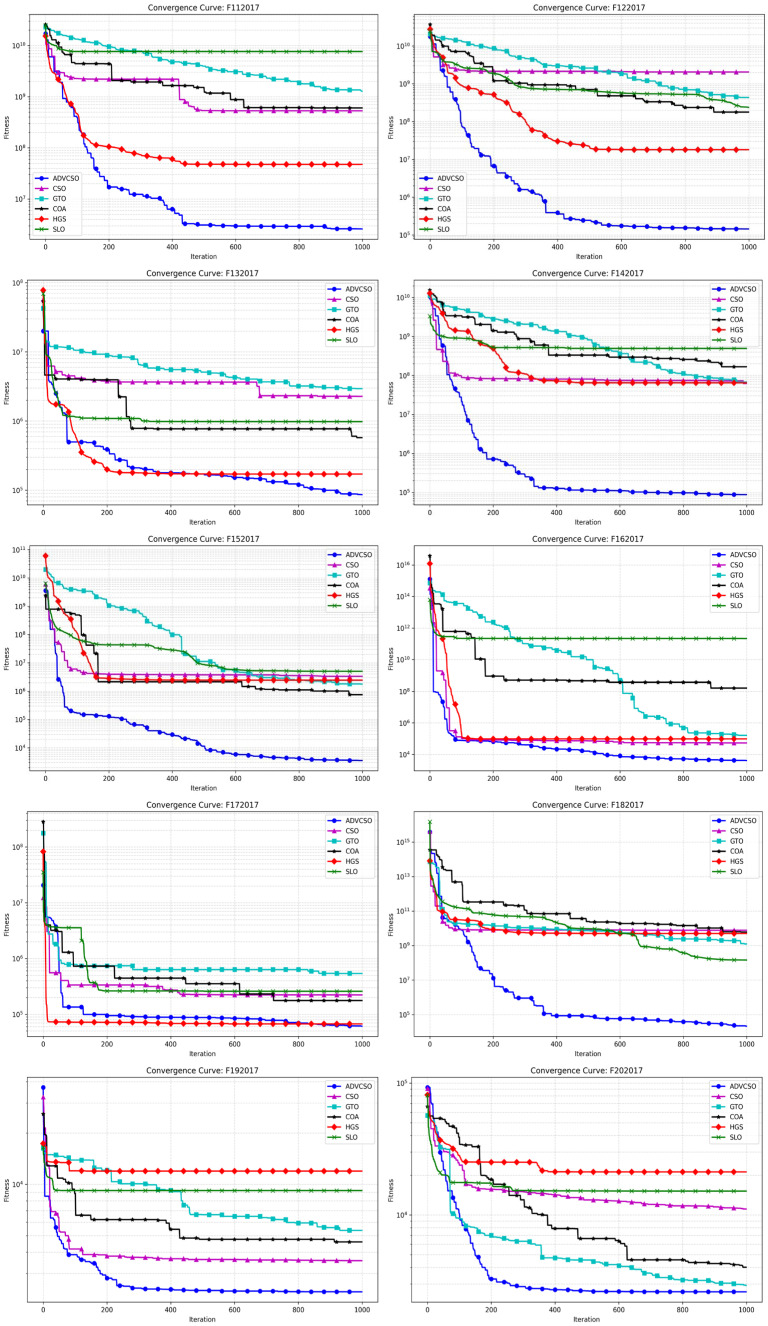

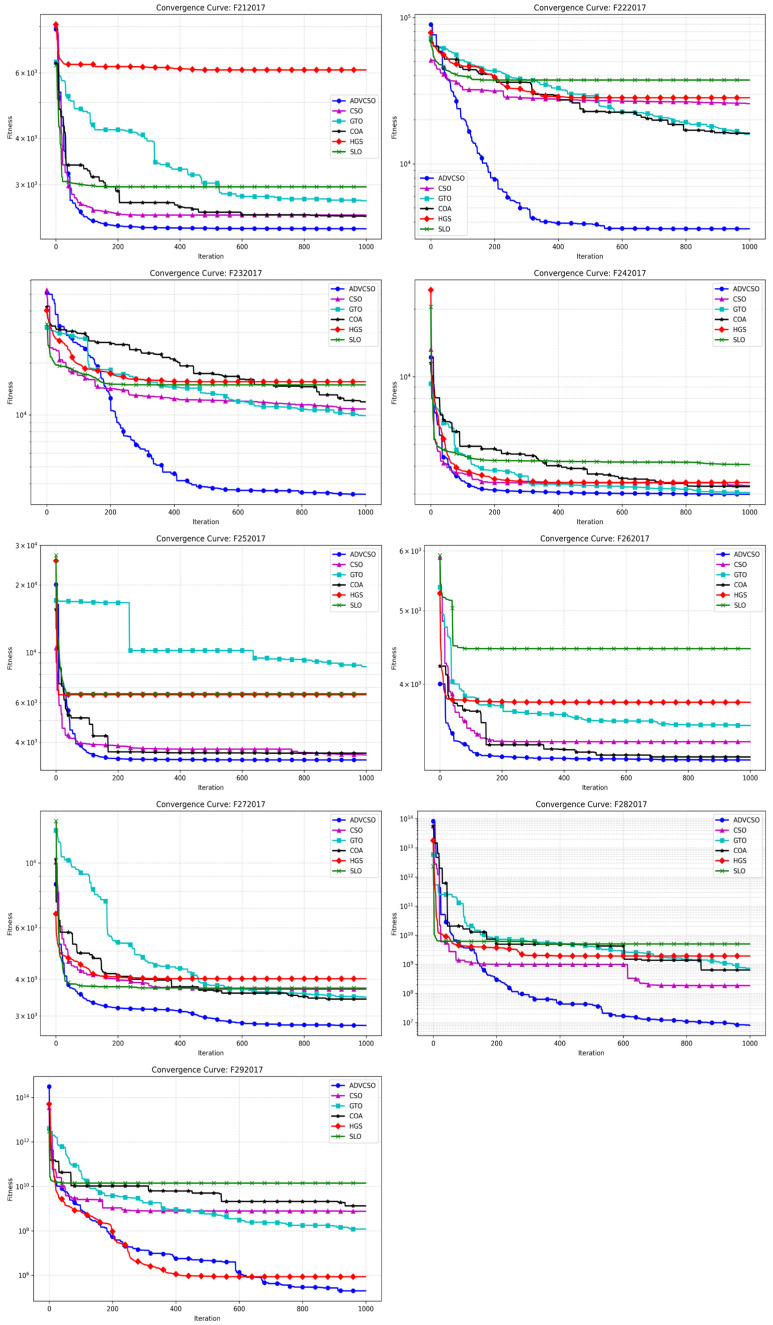
Comparison of convergence curves of various algorithms (1).

Under the aforementioned experimental configuration and algorithmic parameter settings, the ADVCSO and other swarm intelligence algorithms were executed on the CEC2017 test functions, yielding the convergence curves illustrated in Figure 5. Additionally, each algorithm was independently run 50 times, with results summarized in Table 5, including metrics such as mean values and standard deviations.

Figure 5 presents the convergence curve comparison between the ADVCSO and CSO, GTO, COA, HGS, and SLO on the CEC2017 test functions. The results demonstrate that the ADVCSO algorithm achieved the best convergence precision and speed on the vast majority of test functions, ranking first in all cases except F7 and F9. The ADVCSO’s excellent performance on different types of functions stems from the match between its three key improvement strategies and various function characteristics. On the unimodal functions F1 and F2, the ADVCSO’s convergence curves descend rapidly, which is attributable to the high-quality initial solution distribution provided by the good point set initialization strategy, enabling the algorithm to quickly lock onto promising search regions. Furthermore, in complex multimodal environments (such as F11), the ADVCSO exhibited the ability to continuously break through local optima, with convergence curves showing multiple distinct descending plateaus, indicating that the algorithm successfully escaped local optima traps by dynamically adjusting role proportions to balance exploration and exploitation capabilities, thereby avoiding premature convergence and achieving excellent convergence results. In composition functions (such as F22 and F27), which are highly nonlinear functions, the ADVCSO could still effectively avoid local optima when other algorithms stagnated, benefiting from the phased mutation mechanism in the hybrid mutation strategy. After reaching preset iteration stages, the algorithm switches from the Cauchy mutation to the Gaussian mutation, enhancing its refined search capability while maintaining the search intensity.

Table 5 further validates the above analysis results, showing that the ADVCSO outperforms comparison algorithms in terms of mean values and standard deviations for most functions. Specifically, the ADVCSO achieves the lowest mean values across all functions except F7, F9, and F17, underscoring its enhanced convergence precision and stability. Notably, on function F27, the ADVCSO attains a mean value of 2.95 × 10^3^, significantly lower than competitors and approaching the theoretical optimum. Moreover, the ADVCSO exhibits the smallest standard deviations in nearly all test functions, validating its exceptional robustness and consistency. On functions F1, F4, F11, F12, F14, F16, F18, F28, and F29, its standard deviations are significantly lower than that of other comparative algorithms by more than two orders of magnitude.

These results collectively illustrate how the ADVCSO’s enhancement strategies work synergistically under different optimization stages and problem characteristics, enabling the algorithm to intelligently adjust its search behavior, solidifying its efficacy in addressing complex multimodal optimization problems.

**Figure 6:**
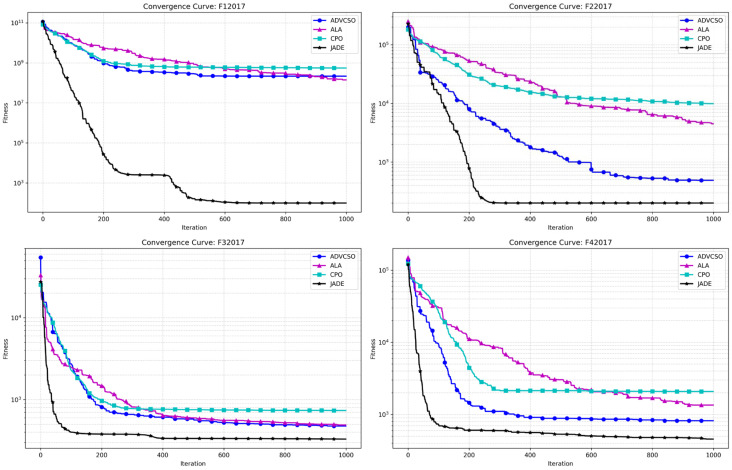

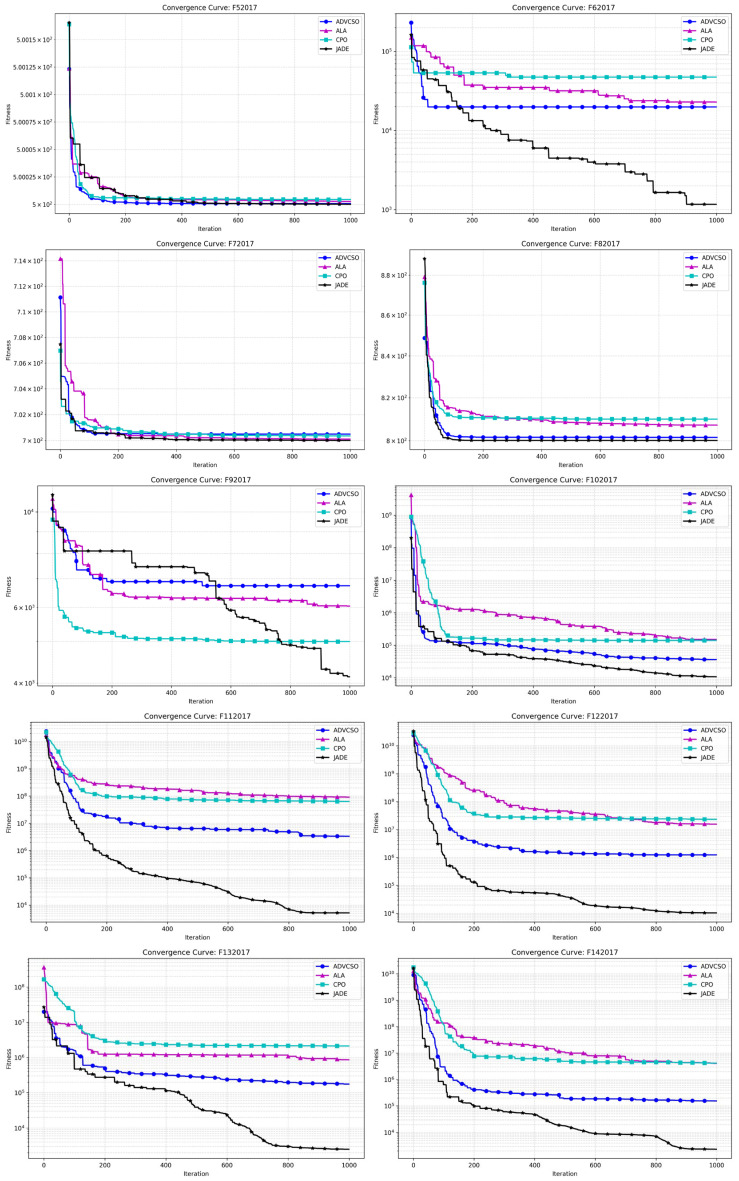

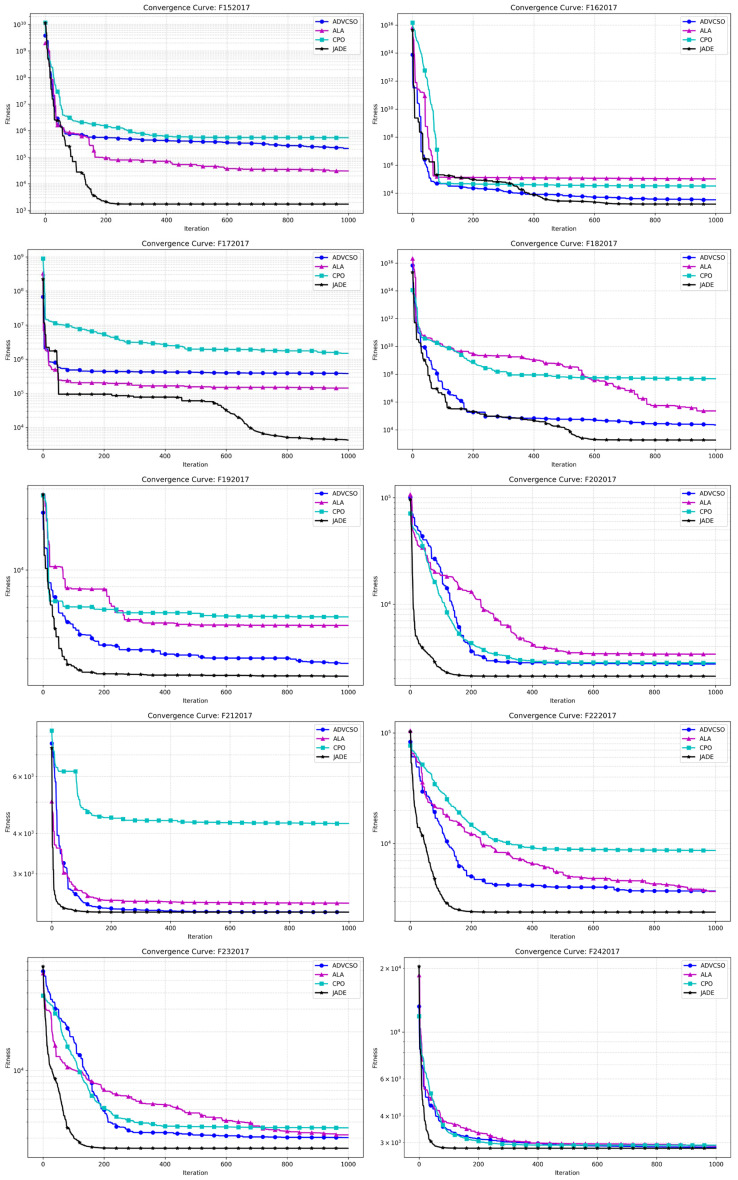

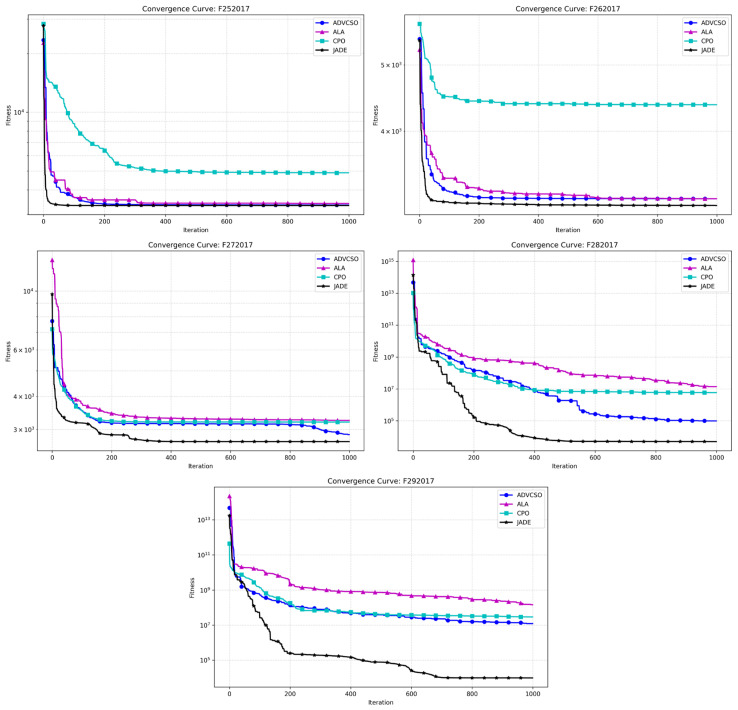
Comparison of convergence curves of various algorithms (2).

To further validate the improvement effects of the ADVCSO, this section also selected recently published state-of-the-art optimization algorithms from high-level journals for comparison, including the Artificial Lemming Algorithm (ALA) [32], Chinese Pangolin Optimizer (CPO) [33], and the CEC champion algorithm JADE [34]. All algorithms were configured with uniform parameters: a problem dimension of 30, a population size of 50, and 1000 iterations. The convergence curves obtained by running the ADVCSO and the aforementioned algorithms on the CEC2017 test function suite are shown in Figure 6. Additionally, each algorithm was independently run 50 times, with results presented in Table 6, which shows metrics such as mean values and standard deviations for each algorithm.

Figure 6 displays the convergence curves of various algorithms on functions F1-F29 from the CEC2017 test function suite. The blue curve representing the ADVCSO in the unimodal function F2 shows a convergence speed slightly inferior to JADE but still significantly superior to the ALA and CPO, ultimately converging to a better fitness value. In the simple multimodal functions F6 and F8, the ADVCSO exhibited a notably faster initial convergence than JADE, demonstrating the powerful advantage of its elite perturbation initialization strategy based on good point sets. Notably, in the composition function F28, all algorithms started with very high initial fitness values, reaching 10^13^–10^15^. The ADVCSO performed similarly to the CPO during the first 400 iterations but showed leap-like improvements between iterations 400 and 600, ultimately converging to 9.83 × 10^4^, significantly outperforming the CPO (5.86 × 10^6^) and ALA (1.39 × 10^7^), demonstrating the effectiveness of the dynamic role allocation mechanism and hybrid mutation strategy improvements.

Table 6 specifically presents the mean values and standard deviations of each algorithm on all 29 functions in the CEC2017 test function suite. The results show that the ADVCSO outperformed the ALA on 23 functions and the CPO on 25 functions. Particularly on the hybrid complex functions F14–F16, the ADVCSO’s average values were 1–3 orders of magnitude lower than the ALA and CPO, indicating that the ADVCSO can effectively reduce the probability of premature convergence and escape local optima traps. Although JADE demonstrated a powerful performance in the experiments, the ADVCSO also showed a strong competitiveness. For example, in most composition functions, such as F21, F24, F25, F26, F27, etc., the ADVCSO’s performance was very close to this champion algorithm, differing by only 10⁻^2^ orders of magnitude, demonstrating its strong adaptability to complex multimodal optimization problems.

The comprehensive experimental results indicate that the ADVCSO algorithm effectively overcomes the deficiencies of the traditional CSO algorithm by introducing an elite perturbation initialization strategy based on good point sets, a dynamic role allocation mechanism, and a hybrid mutation strategy. It significantly outperforms classic algorithms on most test functions and demonstrates high-performance advantages compared to recently published high-level algorithms like the ALA and CPO. Although there remains a certain gap compared to the champion algorithm JADE, the ADVCSO has demonstrated a comparable or even better performance on some complex functions.

### 4.3. Multi-Traveling Salesman Problem Solving

The Multiple Traveling Salesman Problem (MTSP) extends the classical Traveling Salesman Problem (TSP) by scaling the number of salesmen from one to multiple, optimizing the coordinated path planning across cities. In the MTSP, multiple salesmen depart from a central starting city, collaboratively visit all cities, and return to the origin. The objective is to identify a set of routes that minimizes the total distance (or cost) traversed by all salesmen, with the constraint that each city (except the origin) must be visited exactly once [35]. The MTSP finds broad applications in logistics distribution, task allocation, and related fields. The mathematical model of the MTSP is established as follows:

Let m denote the number of salesmen. The decision variable $x_{ij}^{k}\in \left(0,\;1\right)$ indicates whether salesman k travels from city i to city j. The objective function minimizes the maximum path length among all salesmen, formulated as Equation (32):

(32) $$min\left(\underset{1\leq k\leq m}{\max}\sum_{i=0}^{n}\sum_{j=0}^{n}d_{ij}x_{ij}^{k}\right)$$

where $d_{ij}$ represents the Euclidean distance between cities i and j.

The constraints of the MTSP model are as follows:

1. City Visit Uniqueness (Equation (33))

(33) $$\sum_{k=1}^{m}\sum_{j=0}^{n}x_{ij}^{k}=1,i\neq 0$$

2. Route Continuity (Equation (34))

(34) $$\sum_{j=1}^{n}x_{1j}^{k}=1,\sum_{i=1}^{n}x_{i0}^{k}=1$$

A probability-based encoding scheme is adopted to compute path lengths in the MTSP model. For each city $i\left(i\neq 0\right)$, a probability vector $p_{i}=\left[p_{i1},p_{i2},\ldots ,p_{im}\right]\in {\left[0,1\right]}^{m}$ defines the likelihood of assigning i to different salesmen, ensuring $\sum_{k=1}^{m}p_{ik}=1$. The decoding process follows Equations (35)–(38):

(35) $$k_{i}=\underset{1\leq k\leq m}{\max}p_{ik}$$

(36) $$P_{k}=\left\{0\right\}\cup \left\{i\;|\;k_{i}=k\right\}\cup \left\{0\right\}$$

(37) $$L_{k}=\sum_{l=0}^{\left|P_{k}-1\right|}d_{P_{k}\left[l\right],P_{k}\left[l+1\right]}$$

(38) $$f\left(x\right)=\underset{1\leq k\leq m}{max}L_{k}+0.1\sigma_{L}$$

where $k_{i}$ represents the salesman k assigned to city i (highest probability). $P_{k}$ represents the complete route of salesman k. $L_{k}$ represents the total path length of salesman k. $f\left(x\right)$ is the fitness function incorporating the path-balancing penalty (standard deviation of path lengths scaled by weighting factor $\sigma_{L}$).

**Figure 7:**
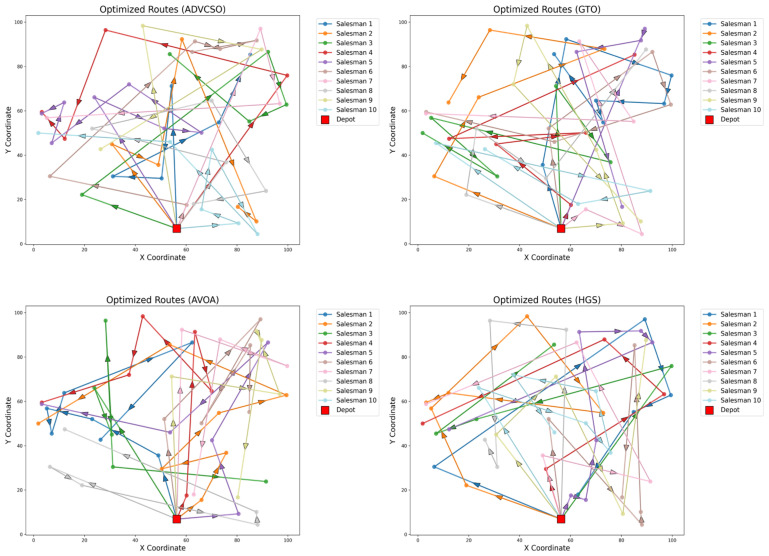
Various algorithms solve the optimal path planning for the MTSP.

In the experiments, 50 randomly generated cities were used, with the distribution center fixed at the first city and 10 salesmen deployed. The proposed ADVCSO was compared against the GTO, African Vultures Optimization Algorithm (AVOA) [36], and HGS. All algorithms were configured with a population size of 50 and 1000 iterations. The optimal path plans for the MTSP obtained by the ADVCSO and other algorithms are illustrated in Figure 7, while statistical results from 20 independent runs are summarized in Table 7.

Figure 7 reveals that the ADVCSO generates paths with a balanced spatial distribution, where each salesman’s assigned region is logically partitioned, avoiding path overlaps and revisits, which underscores the algorithm’s collaborative optimization capability. Table 7 demonstrates the ADVCSO’s superiority across all key metrics: it achieves the best maximum path length (358.27), total path length (1744.09), and fitness value, outperforming the suboptimal algorithms by 6.0% and 5.6% in the maximum and total lengths, respectively. Furthermore, the ADVCSO exhibits the smallest standard deviations among all compared algorithms, confirming its exceptional robustness in combinatorial optimization, even under extreme scenarios.

### 4.4. Multi-Knapsack Problem Solving

The Multiple Knapsack Problem (MKP) extends the classical Knapsack Problem. In the MKP, given a set of items and multiple capacity-constrained knapsacks, the objective is to allocate items to knapsacks to maximize the total value of all items while satisfying each knapsack’s capacity limit [37]. The MKP has broad applications in resource allocation, portfolio optimization, and related fields.

This study employs integer encoding to establish the MKP model. Considering that during the algorithm iteration process, individual position updates may produce infeasible solutions that do not satisfy constraints, the following repair strategies are adopted to ensure all solutions remain within the feasible domain: When single knapsack constraints are violated, such as when an item i is allocated to multiple knapsacks (there exist multiple j where $z_{i}=j$), the allocation with the highest value-to-weight ratio ($\frac{v_{i}}{w_{i}}$) is retained while other allocations are set to zero. When capacity constraints are violated, such as when the total weight of a knapsack j exceeds its capacity $c_{j}$, this study arranges the items in that knapsack in descending order of the value-to-weight ratio ($\frac{v_{i}}{w_{i}}$), then sequentially removes items with the lowest value-to-weight ratio until the capacity constraint is satisfied. Removed items are set to an unassigned state ($z_{i}=0$). Additionally, by introducing penalty terms in the objective function, the feasibility of solutions is ensured, guiding the algorithm toward the feasible domain. The mathematical model of the MKP is established as follows:

Let n denote the number of items with weights $w_{i}$ and values $v_{i}\;\left(i\in \left\{1,\;2,\;\ldots ,\;n\right\}\right)$ and m knapsacks with capacities $c_{j}\;\left(j\in \left\{1,\;2,\;\ldots ,\;m\right\}\right)$. The decision variable $x_{ij}\in \left(0,\;1\right)$ indicates whether item i is placed in knapsack j. The objective function maximizes the total value of the assigned items without exceeding capacities, which is formulated as Equation (39):

(39) $$max\sum_{i=1}^{n}\sum_{j=1}^{m}v_{i}x_{ij}$$

The constraints of the MKP model are as follows:

1. Single-Knapsack Constraint (Equation (40))

(40) $$\sum_{j=1}^{m}x_{ij}\leq 1$$

2. Capacity Constraint (Equation (41))

(41) $$\sum_{i=1}^{n}w_{i}x_{ij}\leq c_{j}$$

An integer encoding scheme is employed to model the MKP. Each individual is encoded as a vector $z=\left[z_{1},z_{2},\ldots ,z_{n}\right]$, where $z_{i}\in \left[0,1,2,\ldots ,m\right]$. $z_{i}=j$ indicates that item i is assigned to knapsack j. The decoding process follows Equations (42)–(46):

(42) $$S_{j}=\left\{i\;|\;z_{i}=j\right\}$$

(43) $$p_{i}=\frac{v_{i}}{w_{i}},\forall i\in S_{j}$$

(44) $$\sum_{i\in S_{j}}w_{i}\leq c_{j}$$

(45) $$j^{*}=\underset{1\leq j\leq m}{\max}\left\{v_{i}|\sum_{k\in S_{j}}{w_{k}+w}_{i}\leq c_{j}\right\},z_{i}=0$$

(46) $$f\left(z\right)=\sum_{i=1}^{n}\sum_{j=1}^{m}v_{i}\delta \left(z_{i},j\right)-1000\cdot \sum_{j=1}^{m}max\left(0,\sum_{i=1}^{n}w_{i}\delta \left(z_{i},j\right)-c_{j}\right)$$

where $S_{j}$ represents a set of items initially assigned to knapsack j. $p_{i}$ represents sorting $S_{j}$ in a descending order of value density and removing items until the capacity constraint is met. $j^{*}$ represents selecting feasible backpacks for unallocated items $i\left(z_{i}=0\right)$. $f\left(z\right)$ is the fitness function of the MKP, penalizing infeasible solutions by returning 0 if any item remains unassigned. An overcapacity penalty is further integrated to enforce a strict adherence to knapsack capacities.

In the experiments, a multi-knapsack instance with 10 items and 3 knapsacks (capacities: 5.3, 4.5, and 6.2) was selected. The weights and values of the items are listed in Table 8, with the maximum total value of the instance being 21.4. The ADVCSO was compared against the GTO, AVOA, and HGS, with all algorithms configured to a population size of 50 and 30 iterations. Each algorithm was executed 20 times, with results summarized in Table 9.

Table 6 shows that the ADVCSO and other algorithms successfully identified the global optimal solution—placing items 4 and 9 in knapsack 1, item 6 in knapsack 2, and items 1, 5, and 8 in knapsack 3—validating their fundamental capability in a feasible solution discovery. However, the ADVCSO demonstrates a significant superiority across critical metrics: the worst fitness, mean fitness, standard deviation, and the number of optimal solutions found. Notably, the ADVCSO achieved the optimal solution 13 out of 20 runs, compared to the HGS’s 8/20—a 62.5% improvement in success rate—confirming its enhanced global search capability and stability in combinatorial optimization.

In summary, the ADVCSO demonstrates significant advantages across various collaborative optimization problems. Comparative analyses against state-of-the-art algorithms reveal that the ADVCSO consistently achieves an optimal convergence precision with faster convergence rates, enhancing exploration capabilities in early stages while avoiding local optima entrapment in later phases, thereby strengthening the exploitation efficacy. Notably, in the MTSP and MKP experiments, the ADVCSO maintains a superior performance and consistency throughout the entire optimization process, validating its efficacy in addressing complex combinatorial optimization challenges.

## 5. Conclusions

To address the universal challenges of balancing global convergence and solution quality in complex combinatorial optimization problems, this study proposes an Adaptive Dynamically Enhanced Chicken Swarm Optimization (ADVCSO) algorithm for solving multi-subproblem collaborative optimization challenges. Inspired by chicken swarm social behaviors, we introduce a population diversity-driven dynamic role allocation mechanism and develop a hybrid mutation strategy, resolving the critical limitations of the original CSO—low flexibility and susceptibility to local optima—while significantly enhancing the performance in complex multimodal problems. Additionally, inspired by the natural distribution patterns of chicken swarms, a good point set initialization strategy is implemented to improve the uniformity and diversity of initial populations, addressing the poor initial solution quality of CSO and accelerating convergence.

Experimental analyses demonstrate that the ADVCSO not only consistently approaches optimal values on benchmark tests but also exhibits an exceptional robustness and stability in real-world combinatorial optimization applications. For the Multi-Traveling Salesman Problem (MTSP), the ADVCSO achieves optimal maximum and total path lengths of 358.27 and 1744.09, respectively. In the Multi-Knapsack Problem (MKP), it attains the global optimum 13 times out of 20 runs, outperforming all competitors across metrics and validating its effectiveness and collaborative optimization capability in practical scenarios.

The ADVCSO represents a paradigm breakthrough in complex combinatorial optimization, particularly in addressing the long-standing technical bottleneck of multi-subproblem coupled optimization, marking an innovative advancement in intelligent optimization methodologies. Future research may focus on enhancing the ADVCSO’s scalability and generalization for diverse dynamic scenarios. Furthermore, integrating deep reinforcement learning into intelligent optimization frameworks could extend its applicability to ultra-large-scale distributed optimization tasks, ensuring efficiency and reliability in real-world applications, such as network optimization and energy scheduling, thereby fostering the co-evolution of theoretical innovation and engineering implementation.

## Figures and Tables

**Table 1:** Summary of CSO enhancement efforts.

Researcher (s)	Year	Initialization Enhancements	Position Update Enhancements		Mutation Strategy Enhancements	
			Internal	External	Pre-Update	Post-Update
Liang et al. [18]	2022			√		√
Deb et al. [19]	2021			√		
Li et al. [20]	2023		√		√	
Nagarajan et al. [21]	2024	√		√		
Afzal et al. [22]	2025		√	√		√
Gamal et al. [23]	2021			√		√
Wang et al. [24]	2021		√		√	
Huang et al. [25]	2024		√			√

**Table 2:** Algorithm variants for ablation studies.

Algorithm Variants	Description
ADVCSO	Complete algorithm combining all three improvement strategies
Original CSO	Original Chicken Swarm Optimization algorithm
CSO_GPS	CSO algorithm using only good point set initialization strategy
CSO_DRA	CSO algorithm using only dynamic role allocation mechanism
CSO_MUT	CSO algorithm using only hybrid mutation strategy
CSO_GPS_DRA	CSO algorithm combining good point set initialization and dynamic role allocation mechanism
CSO_GPS_MUT	CSO algorithm combining good point set initialization and hybrid mutation strategy
CSO_DRA_MUT	CSO algorithm combining dynamic role allocation mechanism and hybrid mutation strategy

**Table 3:** Specific situation of CEC2017 test set.

	No.	Functions	$F_{i}^{*}=F_{i}\left(x^{*}\right)$
Unimodal Functions	1	Shifted and Rotated Bent Cigar Function	100
	2 ^1^	Shifted and Rotated Zakharov Function	200
Simple Multimodal Functions	3	Shifted and Rotated Rosenbrock’s Function	300
	4	Shifted and Rotated Rastrigin’s Function	400
	5	Shifted and Rotated Expanded Scaffer’s F6 Function	500
	6	Shifted and Rotated Lunacek Bi Rastrigin Function	600
	7	Shifted and Rotated Non-Continuous Rastrigin’s Function	700
	8	Shifted and Rotated Levy Function	800
	9	Shifted and Rotated Schwefel’s Function	900
Hybrid Functions	10	Hybrid Function 1 (N = 3)	1000
	11	Hybrid Function 2 (N = 3)	1100
	12	Hybrid Function 3 (N = 3)	1200
	13	Hybrid Function 4 (N = 4)	1300
	14	Hybrid Function 5 (N = 4)	1400
	15	Hybrid Function 6 (N = 4)	1500
	16	Hybrid Function 6 (N = 5)	1600
	17	Hybrid Function 6 (N = 5)	1700
	18	Hybrid Function 6 (N = 5)	1800
	19	Hybrid Function 6 (N = 6)	1900
Composition Functions	20	Composition Function 1 (N = 3)	2000
	21	Composition Function 2 (N = 3)	2100
	22	Composition Function 3 (N = 4)	2200
	23	Composition Function 4 (N = 4)	2300
	24	Composition Function 5 (N = 5)	2400
	25	Composition Function 6 (N = 5)	2500
	26	Composition Function 7 (N = 6)	2600
	27	Composition Function 8 (N = 6)	2700
	28	Composition Function 9 (N = 3)	2800
	29	Composition Function 10 (N = 3)	2900
Search Range: [−100,100]^D 2^			

^1^ The original F2 function in the CEC2017 test suite was officially removed due to defects. ^2^ D denotes the problem dimensionality.

**Table 4:** Algorithm parameters.

Algorithm Name	Parameter	Initial Setting
CSO	Update cycle	5
	The proportion of roosters, hens, and chicks	20%, 30%, and 50%
ADVCSO	Update cycle	Vary with iterations
	The proportion of roosters, hens, and chicks	Vary with iterations
GTO	Position-change-controlling parameter	0.4
	Initial value of jump slope	Random
COA	Number of packs	10
	Number of coyotes per pack	5
	Probability of leaving a pack	0.125
HGS	The probability of updating position	0.08
	Largest hunger/threshold	10,000
SLO	The speed of sound of sea lion leader	Random
AVOA	Probability of status transition in the exploration phase	0.6
	Probability of status transition in phase 1	0.4
	Probability of status transition in phase 2	0.6
	Probability of selecting the 1st best	0.8

**Table 5:** Comparison results of algorithms on the CEC2017 test functions. Significant values are the best results in the comparison of the algorithm performance (1).

Func.	ADVCSO		CSO		GTO		COA		HGS		SLO	
	Mean	Std	Mean	Std	Mean	Std	Mean	Std	Mean	Std	Mean	Std
F1	**1.63E+08**	**3.58E+07**	1.70E+10	3.99E+09	6.83E+09	1.54E+09	9.77E+09	3.34E+09	7.60E+09	3.43E+09	1.14E+10	5.06E+09
F2	**6.39E+02**	**1.00E+02**	3.83E+04	8.01E+03	3.33E+04	3.28E+03	3.29E+04	6.61E+03	3.10E+04	8.46E+03	4.17E+04	1.17E+04
F3	**5.21E+02**	**1.17E+02**	1.67E+03	5.81E+02	8.35E+02	2.00E+02	1.18E+03	2.24E+02	1.18E+03	4.16E+02	3.03E+03	1.74E+03
F4	**8.98E+02**	**7.24E+01**	1.47E+04	4.33E+03	1.21E+04	3.30E+03	1.68E+04	3.84E+03	2.48E+04	6.52E+03	3.07E+04	7.86E+03
F5	**500.0010**	**7.23E−04**	500.0020	1.34E−03	500.0027	2.01E−03	500.0095	2.80E−03	500.0044	3.54E−03	500.0085	6.38E−03
F6	**1.44E+04**	5.64E+03	1.44E+04	**4.55E+03**	2.01E+04	1.04E+04	2.80E+04	9.36E+03	1.04E+04	6.80E+03	1.20E+04	6.87E+03
F7	700.4271	2.32E−01	**700.1991**	**1.43E−01**	700.3934	3.00E−01	700.6601	2.75E−01	700.4914	3.43E−01	700.6061	4.53E−01
F8	**8.05E+02**	**2.36E+00**	8.11E+02	3.68E+00	8.09E+02	2.79E+00	8.09E+02	2.65E+00	8.15E+02	5.78E+00	8.17E+02	5.94E+00
F9	6.56E+03	5.20E+02	6.70E+03	9.70E+02	**5.54E+03**	7.24E+02	6.13E+03	**3.19E+02**	6.36E+03	1.08E+03	6.49E+03	9.12E+02
F10	**3.33E+04**	**1.69E+04**	1.99E+05	2.34E+05	1.20E+05	4.67E+04	5.07E+05	2.13E+05	4.21E+06	2.82E+07	4.12E+05	5.77E+05
F11	**5.30E+06**	**2.08E+06**	1.82E+09	8.00E+08	6.28E+08	2.67E+08	7.24E+08	2.59E+08	1.06E+08	1.74E+08	9.19E+08	1.36E+09
F12	**3.02E+05**	**1.90E+05**	1.56E+09	1.04E+09	4.55E+08	2.71E+08	3.14E+08	1.29E+08	6.02E+07	7.37E+07	6.37E+08	1.01E+09
F13	**5.29E+05**	3.90E+05	2.58E+06	1.84E+06	3.59E+06	1.06E+06	6.70E+05	**3.16E+05**	2.17E+06	2.81E+06	3.69E+06	2.95E+06
F14	**1.22E+05**	**5.98E+04**	2.91E+08	2.22E+08	5.58E+07	4.19E+07	5.88E+07	3.13E+07	1.30E+07	2.99E+07	3.19E+08	5.69E+08
F15	**4.75E+05**	6.41E+05	4.67E+06	5.31E+06	9.85E+05	**4.96E+05**	2.38E+06	1.50E+06	1.27E+06	1.77E+06	2.97E+06	4.88E+06
F16	**3.67E+03**	**8.20E+02**	8.04E+04	1.03E+05	2.39E+07	6.70E+07	2.82E+07	7.16E+07	1.60E+07	1.11E+08	1.84E+11	7.59E+11
F17	2.62E+05	4.33E+05	8.27E+05	1.54E+06	3.10E+06	2.66E+06	3.08E+05	1.06E+05	**1.42E+05**	**8.65E+04**	6.43E+05	9.66E+05
F18	**2.22E+05**	**2.67E+05**	1.68E+10	1.61E+10	5.02E+09	6.25E+09	3.04E+09	1.99E+09	8.29E+08	1.60E+09	7.44E+09	1.99E+10
F19	**2.33E+03**	**2.70E+02**	3.85E+03	5.51E+02	6.12E+03	1.34E+03	4.04E+03	4.09E+02	1.04E+04	3.07E+03	7.83E+03	2.31E+03
F20	**2.56E+03**	**1.23E+02**	8.42E+03	3.21E+03	5.98E+03	2.41E+03	4.38E+03	1.12E+03	1.35E+04	9.46E+03	2.05E+04	8.42E+03
F21	**2.28E+03**	**7.69E+00**	2.49E+03	7.71E+01	2.63E+03	1.94E+02	2.42E+03	2.62E+01	3.83E+03	7.47E+02	3.44E+03	5.94E+02
F22	**3.95E+03**	**2.30E+02**	2.03E+04	3.35E+03	1.37E+04	4.26E+03	1.48E+04	2.35E+03	2.12E+04	1.03E+04	3.18E+04	8.87E+03
F23	**3.09E+03**	**2.10E+02**	1.33E+04	2.87E+03	8.05E+03	3.01E+03	1.01E+04	1.63E+03	1.60E+04	6.75E+03	1.79E+04	5.46E+03
F24	**2.87E+03**	**2.40E+01**	3.38E+03	2.11E+02	3.17E+03	7.87E+01	3.21E+03	1.49E+02	3.31E+03	2.08E+02	4.23E+03	8.09E+02
F25	**3.34E+03**	**1.01E+01**	3.67E+03	1.68E+02	5.54E+03	1.46E+03	3.45E+03	7.98E+01	4.99E+03	1.67E+03	7.39E+03	2.58E+03
F26	**3.18E+03**	3.33E+01	3.30E+03	6.24E+01	3.72E+03	3.33E+02	3.23E+03	**2.89E+01**	3.64E+03	2.51E+02	3.97E+03	3.14E+02
F27	**2.95E+03**	1.82E+02	3.55E+03	1.61E+02	3.45E+03	4.01E+02	3.50E+03	**6.71E+01**	3.57E+03	5.45E+02	4.58E+03	9.38E+02
F28	**7.32E+05**	**1.60E+06**	1.04E+09	9.44E+08	3.20E+08	1.94E+08	1.09E+09	7.76E+08	2.56E+08	3.97E+08	1.11E+10	3.63E+10
F29	**3.87E+06**	**6.82E+06**	2.04E+09	1.28E+09	4.45E+08	2.83E+08	9.03E+08	5.23E+08	2.69E+08	2.98E+08	2.98E+09	3.28E+09

**Table 6:** Comparison results of algorithms on the CEC2017 test functions. Significant values are the best results in the comparison of the algorithm performance (2).

Func.	ADVCSO		ALA		CPO		JADE	
	Mean	Std	Mean	Std	Mean	Std	Mean	Std
F1	1.61E+08	5.22E+07	1.90E+08	7.50E+07	4.99E+08	2.42E+08	**1.00E+02**	**7.89E−06**
F2	6.58E+02	1.08E+02	3.90E+03	1.53E+03	1.56E+04	5.37E+03	**2.00E+02**	**2.41E−14**
F3	5.10E+02	1.19E+02	5.12E+02	6.90E+01	5.49E+02	7.66E+01	**3.33E+02**	**1.02E+01**
F4	8.85E+02	7.57E+01	1.22E+03	1.72E+02	2.17E+03	9.48E+02	**4.54E+02**	**7.96E+00**
F5	500.0011	7.67E−04	500.0043	3.64E−03	500.0043	1.13E−02	**500.0007**	**4.75E−05**
F6	1.39E+04	4.15E+03	2.40E+04	1.20E+04	4.53E+04	1.07E+04	**1.91E+03**	**1.14E+03**
F7	700.4619	2.16E−01	700.6509	5.15E−01	700.3131	1.62E−01	**700.0269**	**2.15E−02**
F8	8.04E+02	2.03E+00	8.13E+02	5.00E+00	8.12E+02	6.24E+00	**8.00E+02**	**4.69E−01**
F9	6.63E+03	4.93E+02	6.06E+03	7.78E+02	5.31E+03	3.71E+02	**4.47E+03**	**3.64E+02**
F10	3.25E+04	2.27E+04	2.20E+05	9.80E+04	1.98E+05	4.22E+05	**1.82E+03**	**2.32E+03**
F11	6.13E+06	3.12E+06	9.59E+07	8.24E+07	6.16E+07	4.91E+07	**8.22E+03**	**6.28E+03**
F12	3.38E+05	1.49E+05	1.11E+07	4.84E+06	1.28E+07	8.73E+06	**5.12E+03**	**4.31E+03**
F13	6.93E+05	5.71E+05	1.78E+06	1.24E+06	2.68E+06	1.90E+06	**1.21E+04**	**1.84E+04**
F14	1.34E+05	9.26E+04	3.99E+06	2.31E+06	4.56E+06	2.69E+06	**7.63E+03**	**5.04E+03**
F15	5.76E+05	6.00E+05	6.97E+05	8.49E+05	4.14E+05	5.49E+05	**1.81E+03**	**2.85E+02**
F16	3.73E+03	1.12E+03	1.02E+05	4.80E+04	3.81E+06	2.61E+07	**2.07E+03**	**3.80E+02**
F17	3.39E+05	4.69E+05	5.26E+05	7.55E+05	1.05E+06	1.09E+06	**9.47E+03**	**1.26E+04**
F18	2.09E+05	2.50E+05	1.40E+07	8.52E+07	7.58E+08	2.43E+09	**4.24E+03**	**8.20E+03**
F19	2.36E+03	3.13E+02	4.56E+03	1.04E+03	9.08E+03	2.21E+03	**2.08E+03**	**1.59E+02**
F20	2.57E+03	9.96E+01	2.72E+03	3.29E+02	3.47E+03	6.59E+02	**2.21E+03**	**7.70E+01**
F21	2.28E+03	8.38E+00	2.38E+03	3.57E+01	4.08E+03	8.69E+02	**2.27E+03**	**5.25E+00**
F22	3.86E+03	2.73E+02	3.76E+03	3.31E+02	8.46E+03	2.50E+03	**2.42E+03**	**5.97E+01**
F23	3.13E+03	2.27E+02	3.39E+03	1.50E+02	4.27E+03	5.45E+02	**2.50E+03**	**2.19E+01**
F24	2.86E+03	3.26E+01	2.97E+03	6.82E+01	3.04E+03	7.51E+01	**2.82E+03**	**1.09E−01**
F25	3.34E+03	1.39E+01	3.41E+03	5.98E+00	5.96E+03	1.68E+03	**3.33E+03**	**6.95E−01**
F26	3.19E+03	3.90E+01	3.21E+03	5.18E+01	3.82E+03	4.40E+02	**3.11E+03**	**1.20E+01**
F27	2.91E+03	1.63E+02	3.20E+03	**6.08E+01**	3.42E+03	5.12E+02	**2.75E+03**	7.48E+01
F28	4.42E+05	1.23E+06	1.37E+08	3.46E+08	1.26E+08	4.22E+08	**6.69E+03**	**1.80E+03**
F29	2.96E+06	4.84E+06	5.90E+07	7.78E+07	1.03E+08	1.72E+08	**1.08E+04**	**5.20E+03**

**Table 7:** Comparison results of algorithms for solving the MTSP. Significant values are the best results in the comparison of the algorithm performance.

	ADVCSO		GTO		AVOA		HGS	
	Mean	Std	Mean	Std	Mean	Std	Mean	Std
Max Distance	**358.27**	**21.02**	381.28	21.13	388.96	28.60	454.45	24.25
Total Distance	**1744.09**	**89.33**	1847.04	112.36	1898.86	136.45	2124.22	137.56
Fitness	**359.10**	21.37	382.24	**21.12**	389.74	28.65	456.96	24.46

**Table 8:** Value and weight of items.

Items	1	2	3	4	5	6	7	8	9	10
Value	+2.3	+1.5	+3.4	+1.6	+5.2	+4.3	+2.8	+3.9	+4.1	+2.5
Weight	1.2	3.4	2.5	1.6	1.9	4.3	5.1	2.8	3.5	4.2
Search Range: [0, 21.4]										

**Table 9:** Comparison results of algorithms for solving the MKP. Significant values are the best results in the comparison of the algorithm performance.

	ADVCSO	GTO	AVOA	HGS
Best Fitness	**21.40**	21.40	21.40	21.40
Worst Fitness	**20.90**	20.70	20.70	20.70
Mean Fitness	**21.22**	20.92	21.04	21.07
Standard Deviation	**0.24**	0.29	0.27	0.28
Number of Optimal Solutions Found	**13**	5	7	8

## Data Availability

The data used to support the findings of this study are included within the article and are also available from the corresponding authors upon request. All code is available at https://github.com/du5-05/ADVCSO.git (accessed on 24 April 2025).

## Abbreviations

The following abbreviations are used in this manuscript:

ADVCSO	Adaptive Dynamically Enhanced Variant of Chicken Swarm Optimization
CSO	Chicken Swarm Optimization
SO	Snake Optimizer
DRA	Divine Religions Algorithm
APO	Artificial Protozoa Optimizer
RBMO	Red-Billed Blue Magpie Optimizer
HOA	Hiking Optimization Algorithm
SDO	Sled Dog-Inspired Optimizer
GJO	Golden Jackal Optimization
BOA	Butterfly Optimization Algorithm
SSA	Sparrow Search Algorithm
SCSO	Sand Cat Swarm Optimization
HBA	Honey Badger Algorithm
BES	Bald Eagle Search Algorithm
PECSO	Improved Enhanced Chicken Swarm Algorithm
AFSA	Artificial Fish Swarm Algorithm
GA	Genetic Algorithm
FCSO	Adaptive Fuzzy Chicken Swarm Optimization
NSCSO	Non-Dominated Sorting Chicken Swarm Algorithm
MTSP	Multi-Traveling Salesman Problem
TSP	Traveling Salesman Problem
MKP	Multi-Knapsack Problem
GTO	Giant Trevally Optimizer
COA	Coyote Optimization Algorithm
HGS	Hunger Games Search
SLO	Sea Lion Optimization Algorithm
AVOA	African Vultures Optimization Algorithm
ALA	Artificial Lemming Algorithm
CPO	Chinese Pangolin Optimizer
JADE	Adaptive Differential Evolution with Optional External Archive
